# Real-world experience of circulating tumor DNA testing in resectable colorectal cancer: a Japanese single-institution observational study

**DOI:** 10.1007/s10147-025-02943-7

**Published:** 2025-12-15

**Authors:** Yuko Fukumoto, Kozo Kataoka, Yoshiko Muroi, Mizuki Koba, Kazuma Ito, Rao Zhenxin, Ayako Imada, Song Jihyung, Yuki Horio, Ryuichi Kuwahara, Motoi Uchino, Kei Kimura, Eiji Oki, Masataka Ikeda

**Affiliations:** 1 Division of Lower GI Surgery, Department of Gastroenterological Surgery, Hyogo Medical University, Nishinomiya, Japan; 2 Cancer Center, Hyogo Medical University Hospital, Nishinomiya, Japan; 3 Division of Inflammatory Bowel Disease, Department of Gastroenterological Surgery Hyogo Medical University, Nishinomiya, Japan; 4 Department of Advanced Medicine and Innovative Technology, Kyushu University Hospital, Fukuoka, Japan

**Keywords:** CtDNA, Colorectal cancer, MRD, Real-world data

## Abstract

**Background:**

Assessment of molecular residual disease (MRD) using circulating tumor DNA (ctDNA) is a powerful prognostic tool for detecting postoperative recurrence in colorectal cancer (CRC). However, ctDNA-based MRD testing has been available only within clinical trials in Japan, and its clinical utility in patients ineligible for trials due to age or comorbidities remains unclear. We conducted a prospective observational study to describe the real-world implementation and clinical findings of postoperative ctDNA testing in CRC.

**Methods:**

CRC patients who underwent tumor-agnostic ctDNA MRD testing after curative-intent resection were prospectively enrolled. When ctDNA was detected, early imaging was performed to assess recurrence. Clinical outcomes were analyzed according to ctDNA status.

**Results:**

56 CRC patients who underwent ctDNA testing 4–8 weeks after surgery between June 2023 and June 2025 were analyzed. 18 (32.1%) were ctDNA-positive and 38 (67.9%) were ctDNA-negative. Radiological recurrence occurred in 10 of 16 evaluable ctDNA-positive patients (62.5%), including liver metastases in 4 and no lung metastases. In contrast, recurrence was observed in 5 of 37 ctDNA-negative patients (13.5%), including lung metastases in 3 and no liver metastases. Three ctDNA-positive patients (18.8%) achieved ctDNA clearance after adjuvant chemotherapy and remained recurrence-free, whereas persistent-ctDNA positivity predicted disease progression. In the ctDNA-negative cohort, 84.5% remained disease-free regardless of adjuvant therapy.

**Conclusions:**

This interim report demonstrates the feasibility of implementing postoperative ctDNA testing in real-world clinical practice. While exploratory and descriptive in nature, the findings suggest that ctDNA status may reflect recurrence risk and provide useful information for postoperative management in resectable CRC.

## Introduction

Colorectal cancer (CRC) is the third leading cause of cancer-related death worldwide [1]. Although substantial advances have been achieved in surgical techniques and adjuvant therapies, approximately 80% of patients with stage II, 50% with stage III, and 30% with stage IV disease can be cured by surgery alone [2–4]. Adjuvant chemotherapy (ACT) is generally recommended for patients with stage III and selected stage II and IV patients [5–7]. However, it is difficult to predict who is most likely to benefit from ACT due to lack of reliable biomarkers to identify those at high risk of recurrence. Recent studies have increasingly highlighted the utility of circulating tumor DNA (ctDNA) as a biomarker for detecting molecular residual disease (MRD) following CRC resection [8–12]. Across all stages of resectable CRC, postoperative ctDNA-positive patients exhibit significantly lower disease-free survival (DFS) and overall survival (OS) compared to ctDNA-negative patients. Furthermore, analyses focusing on high-risk stage II and stage III CRC indicate that among ctDNA-positive patients, those receiving ACT have significantly improved DFS compared to those who did not. Conversely, no significant difference in DFS has been observed between ctDNA-negative patients who did or did not receive ACT. These findings indicate that postoperative ctDNA assessment could serve as an informative tool for guiding decisions on adjuvant chemotherapy.

However, most reports reflect the findings of clinical research with only a few randomized clinical trials, and the real-world applicability of ctDNA testing in routine clinical practice remains undetermined in Japan. At our institution, postoperative ctDNA testing for resectable CRC has been offered on a self-funded basis since June 2023 and data from patients who have undergone ctDNA testing are being accumulated in a prospective observational study. The aim of this study is to clarify how this can be effectively utilized in real-world clinical settings, particularly for elderly patients, those with poor performance status, and those with severe comorbidities such as chronic kidney disease. Herein, we report the interim findings of this ongoing observational cohort.

## Methods

### Patient cohort

This is a prospective observational study conducted at Hyogo medical university, aiming to describe the real-world implementation and clinical use of ctDNA testing in Japan. Patients who underwent curative surgical resection and chose to receive ctDNA testing were enrolled after providing written informed consent. Since this study focuses on real-world feasibility, testing patterns, and early clinical insights rather than inferential statistical comparisons, no formal hypothesis testing or sample size calculation based on statistical power was performed. The primary endpoint is ctDNA negativity as a marker of molecular residual disease, and the planned total sample size is 100. The patient accrual and follow-up periods are 3 and 5 years, respectively. This study commenced in June 2023.

In this interim report, patients with resectable CRC who received tumor-agnostic ctDNA MRD testing 4 to 10 weeks after curative-intent surgery at Hyogo medical university between June 2023 and June 2025 were analyzed. When ctDNA MRD was present, follow-up imaging was conducted within one month of the report to assess early recurrence. Computed tomography (CT) was performed every 6 months after surgery according to the Japanese society of cancer of the colon and rectum (JSCCR) guidelines [13]. Treatment decisions were based on standard clinical judgment primarily considering tumor stage and patient background, including age, performance status, and comorbidities. Results of ctDNA testing were considered supplemental. For example, ctDNA-positive patients could be offered intensification of adjuvant therapy, e.g., extending CAPOX from 3 to 6 months. On the other hand, ctDNA-negative patients could be considered for treatment de-escalation, such as switching from CAPOX to capecitabine monotherapy or shortening CAPOX duration. The final treatment plan was determined through shared decision-making between the treating physician and the patient.

### Ethical consideration and billing status of ctDNA

ctDNA testing was performed on a self-pay basis as a non-reimbursed service. All procedures covered by national health insurance, such as imaging examinations and adjuvant chemotherapy, were conducted according to standard clinical indications and at the discretion of the treating physicians. The ctDNA results did not prompt medical services beyond those currently recommended and reimbursed. This study was conducted in accordance with the declaration of Helsinki and written informed consent was obtained from all study participants prior to participation. The protocol was approved by the institutional review board of the Hyogo university hospital (No. 4526). All aspects of the study design, data collection, data management, and analysis were conducted independently by the investigators at Hyogo medical university. Guardant health had no access to identifiable data and was not involved in data analysis or interpretation. Statistical review and interpretation were performed solely by the investigators at Hyogo medical university.

### MRD analysis

MRD analysis was performed using the tissue-agnostic guardant reveal assay [14]. Up to 30 ng of cell-free DNA extracted from 2 to 5 mL of plasma was partitioned based on its methylation state. Next-generation sequencing data were analyzed using proprietary bioinformatic software trained to detect the presence of ctDNA based on epigenomic signals from ∼2000 differentially methylated regions (DMR) strongly associated with colorectal cancer. Each sample was classified as ctDNA detected (positive) or not detected (negative). Tumor fraction was estimated by normalizing cancer-specific DMR with appropriately matched control regions within each sample.

## Results

### Patient characteristics

**Fig. 1:**
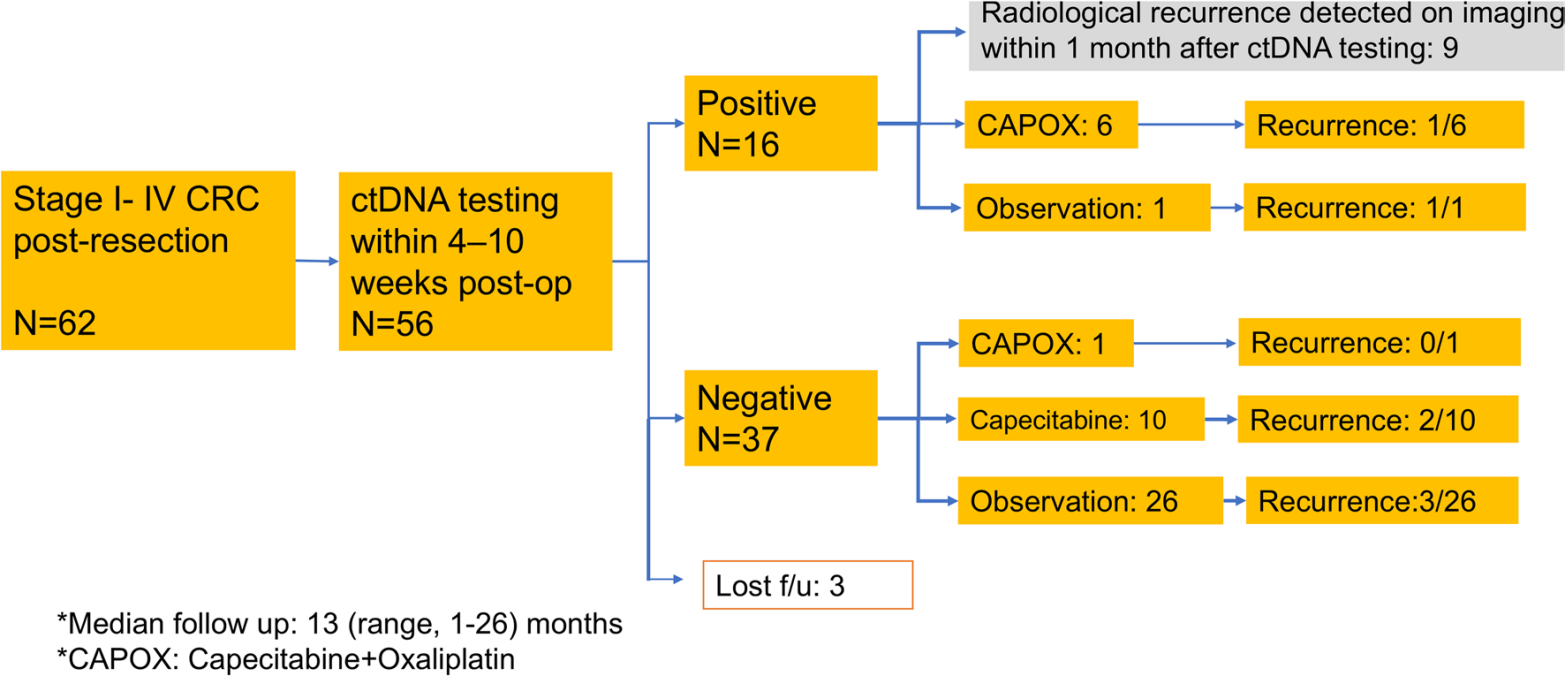
Patient flow diagram

A total of 62 patients underwent ctDNA analysis following colorectal cancer surgery between June 2023 and June 2025. Among them, 56 patients had ctDNA evaluated within 4 to 10 weeks postoperatively, with median turnaround time from blood collection to ctDNA result was 9 (range 6–13) days. All samples were successfully analyzed, resulting in 100% quality control pass rate. Of these 56 patients, 18 were ctDNA-positive and 38 were ctDNA-negative. Median follow-up was 13 (range 1–26) months. Three patients (2 ctDNA-positive and 1 ctDNA-negative) were censored due to follow-up at external institutions (Fig. 1). When ctDNA was detected, additional imaging examinations (CT scan, liver MRI and PET if possible) were performed to detect early recurrence. Patient characteristics of the 53 patients excluding 3 patients who were lost to follow-up are detailed in Table 1. Most patients had an ECOG performance status of 0; however, approximately 60% had one or more comorbidities.

**Table 1:** Patient characteristics

	Characteristic	All patients (*n* = 53)	ctDNA(−) (*n* = 37)	ctDNA(+) (*n* = 16)
Age	Median, range	69 (33–84)	70 (40–84)	59 (33–83)
Sex	Male	32 (60.4%)	22 (59.5%)	10 (62.5%)
	Female	21 (39.6%)	15 (40.5%)	6 (37.5%)
Location of primary tumor	Right colon	20 (37.7%)	15 (40.5%)	5 (31.3%)
	Left colon	14 (26.4%)	7 (18.9%)	7 (43.8%)
	Rectum	19 (35.8%)	15 (40.5%)	4 (25.0%)
Duration between ctDNA testing and surgery	days	32 (27–71)	32 (27–71)	34 (28–49)
pStage	I	4 (7.5%)	3 (8.1%)	1 (6.3%)
	II	8 (15.1%)	8 (21.6%)	0 (0.0%)
	III	28 (52.8%)	19 (51.4%)	9 (56.3%)
	IV	13 (24.5%)	7 (18.9%)	6 (37.5%)
RAS	Wild-type	19 (35.8%)	14 (37.8%)	5 (31.3%)
	Mutant	22 (41.5%)	14 (37.8%)	8 (50.0%)
	Unknown	12 (22.6%)	9 (24.3%)	3 (18.8%)
BRAF	Wild-type	37 (69.8%)	25 (67.6%)	12 (75.0%)
	Mutant	4 (5.7%)	3 (8.1%)	1 (6.3%)
	Unknown	12 (28.3%)	9 (24.3%)	3 (18.8%)
MMR	pMMR	35 (66.0%)	25 (67.6%)	10 (62.5%)
	dMMR	3 (5.7%)	2 (5.4%)	1 (6.3%)
	Unknown	15 (28.3%)	10 (27.0%)	5 (31.3%)
Adjuvant chemotherapy (ACT)	CAPOX	7 (13.2%)	1 (2.7%)	6 (37.5%)
	Capecitabine	10 (18.9%)	10 (27.0%)	0 (0.0%)
	None	36 (67.9%)	26 (70.3%)	10* (62.5%)
ECOG performance status	0	51 (96.2%)	36 (97.3%)	15 (93.8%)
	1	2 (3.8%)	1 (2.7%)	1 (6.3%)
	2–4	0	0	0
Comorbidities	Any	33 (62.2%)	25 (67.6%)	8 (50.0%)
	Chronic kidney disease	3 (5.7%)	1 (2.7%)	2 (12.5%)
	Hypertension	22 (41.5%)	16 (43.2%)	6 (37.5%)
	Diabetes mellitus	8 (15.1%)	7 (18.9%)	1 (6.3%)
	Atrial fibrillation	3 (5.7%)	2 (5.4%)	1 (6.3%)
	Stroke	6 (11.3%)	5 13.5%)	1 (6.3%)

*Nine of 10 patients did not receive ACT because metastatic lesions were detected on imaging scans

#### Details of ctDNA-positive cases (*N* = 16)

**Fig. 2:**
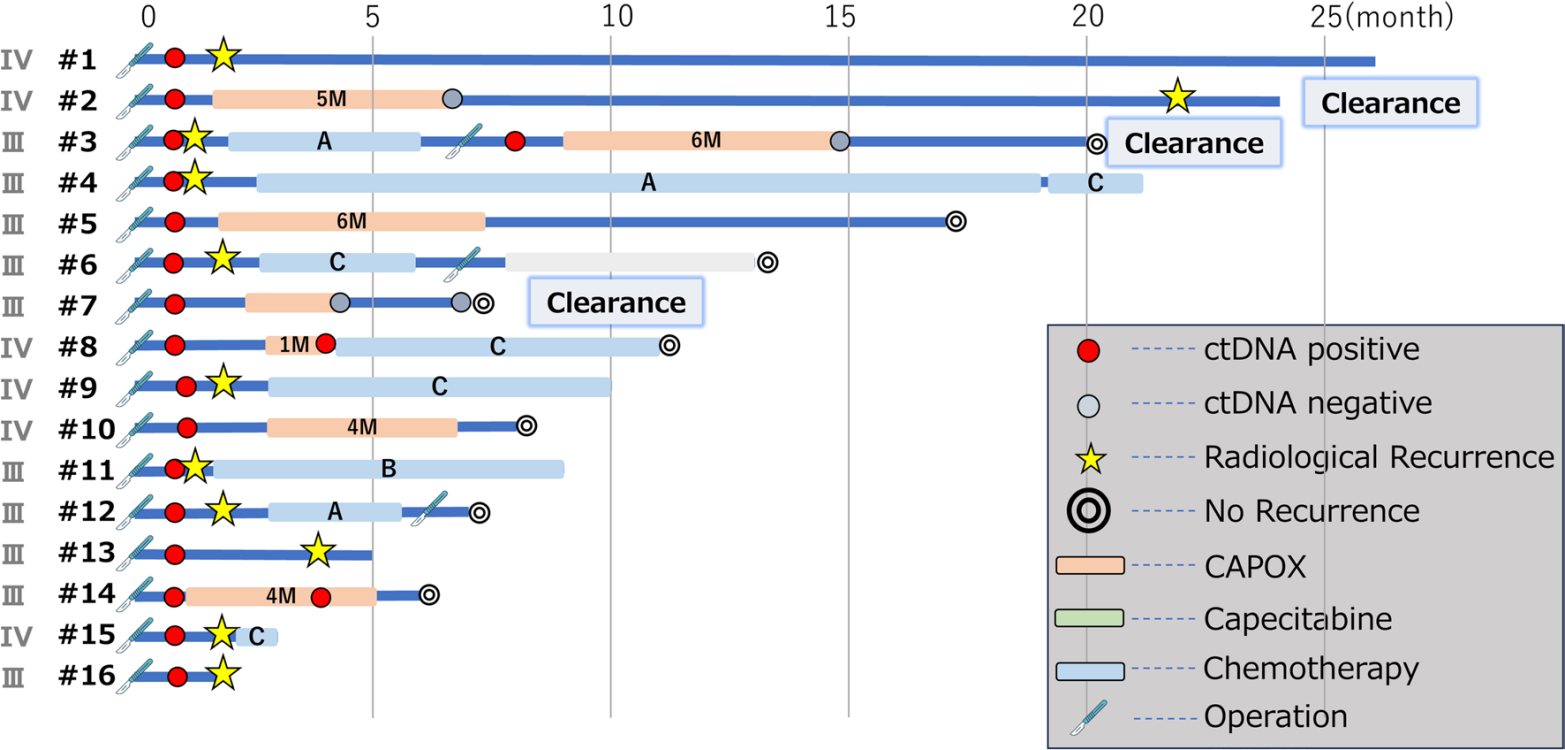
Details of ctDNA-positive patients (*n* = 16). The start point for each bar in the swimmer’s plot corresponds to the date of curative-intent surgery. *A 5-fluorouracil, leucovorin, oxaliplatin, and irinotecan combined with bevacizumab (FOLFOXIRI + Bmab), **B** 5-fluorouracil, leucovorin, and irinotecan combined with bevacizumab (FOLFIRI + Bmab), **C** 5-fluorouracil, leucovorin, and irinotecan combined with ramucirumab (FOLFIRI + Rmab)

Among the 16 patients, median follow-up duration was 9.5 months (range 1–26 months). Imaging within one month of the assay result detected radiological recurrence in 9 patients. The postoperative treatment courses of the 16 patients with ctDNA positivity are summarized in Fig. 2. Among the 9 patients with early recurrence, metastatic sites included the liver (*n* = 4), lymph nodes (*n* = 4), and peritoneum (*n* = 1). All of them received systemic chemotherapy for metastatic disease following the diagnosis of recurrence and three successfully underwent curative metastasectomy. The remaining 6 patients received CAPOX as adjuvant chemotherapy (pStage III/IV: 3/3), with a median follow-up duration of 9.5 months (range 6–24 months). To date, 5 patients (83.3%) remain disease-free and 1 patient (16.7%) had thoracic spine metastasis 22 months after curative primary surgery (#2 in Fig. 2). In contrast, 1 patient (pStage IV) who did not receive adjuvant chemotherapy developed peritoneal recurrence at 4 months postoperatively and was transitioned to best supportive care (#1 in Fig. 2).

#### Surveillance of ctDNA-negative cases

Although ctDNA testing after colorectal cancer surgery is available through self-funding at our institution, repeat testing was performed in some cases upon patient request. Among the 16 patients with available follow-up, 3 underwent serial ctDNA assessments (#2, 3, and 7 in Fig. 2). In cases #2 and #7, imaging studies were performed immediately following the postoperative ctDNA-positive results, but no overt recurrent lesions were identified. However, due to a high suspicion of subclinical micrometastases, both patients received adjuvant CAPOX chemotherapy. Subsequent ctDNA testing demonstrated conversion to ctDNA negativity. Case #2 experienced bone metastasis at 22 months postoperatively and underwent palliative radiotherapy. In contrast, case #7 has remained recurrence-free to date. In case #3, imaging performed based on the postoperative ctDNA-positive result revealed liver metastasis, and the patient received one cycle of FOLFOXIRI plus bevacizumab, followed by surgical resection of the metastatic lesion. ctDNA remained positive one month after the hepatectomy despite the absence of radiological recurrence. Therefore, the patient underwent seven cycles of CAPOX chemotherapy. A third ctDNA test following completion of CAPOX confirmed conversion to negativity, and the patient remains disease-free to date.

#### Tumor fraction and metastatic sites

**Fig. 3:**
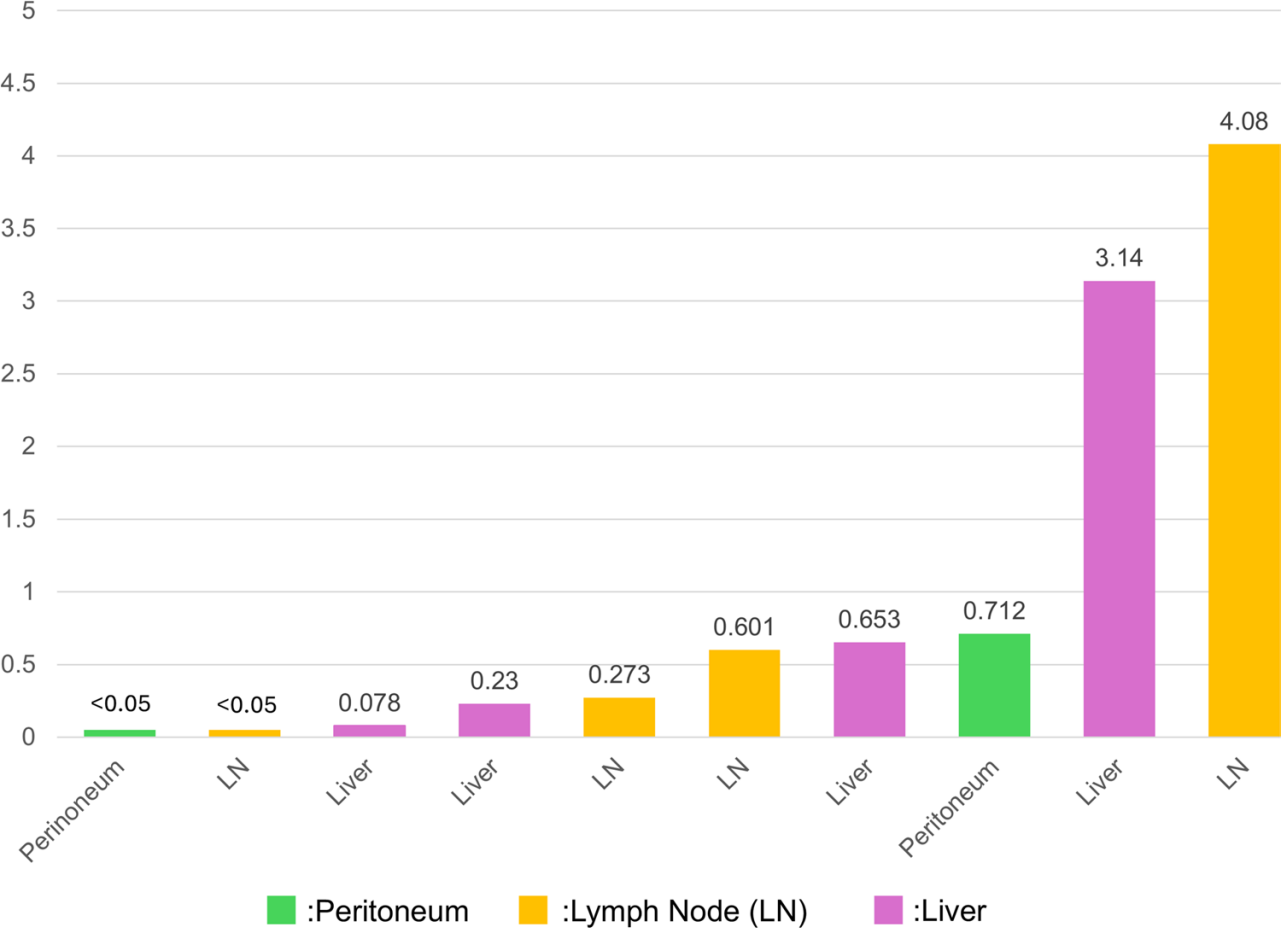
Tumor Fraction and metastatic sites

Figure 3 shows the association between tumor fraction values in ctDNA-positive patients after curative-intent surgery and the sites of recurrence, which were mostly liver and lymph nodes.

### Details of ctDNA-negative cases (*N* = 37)

**Fig. 4:**
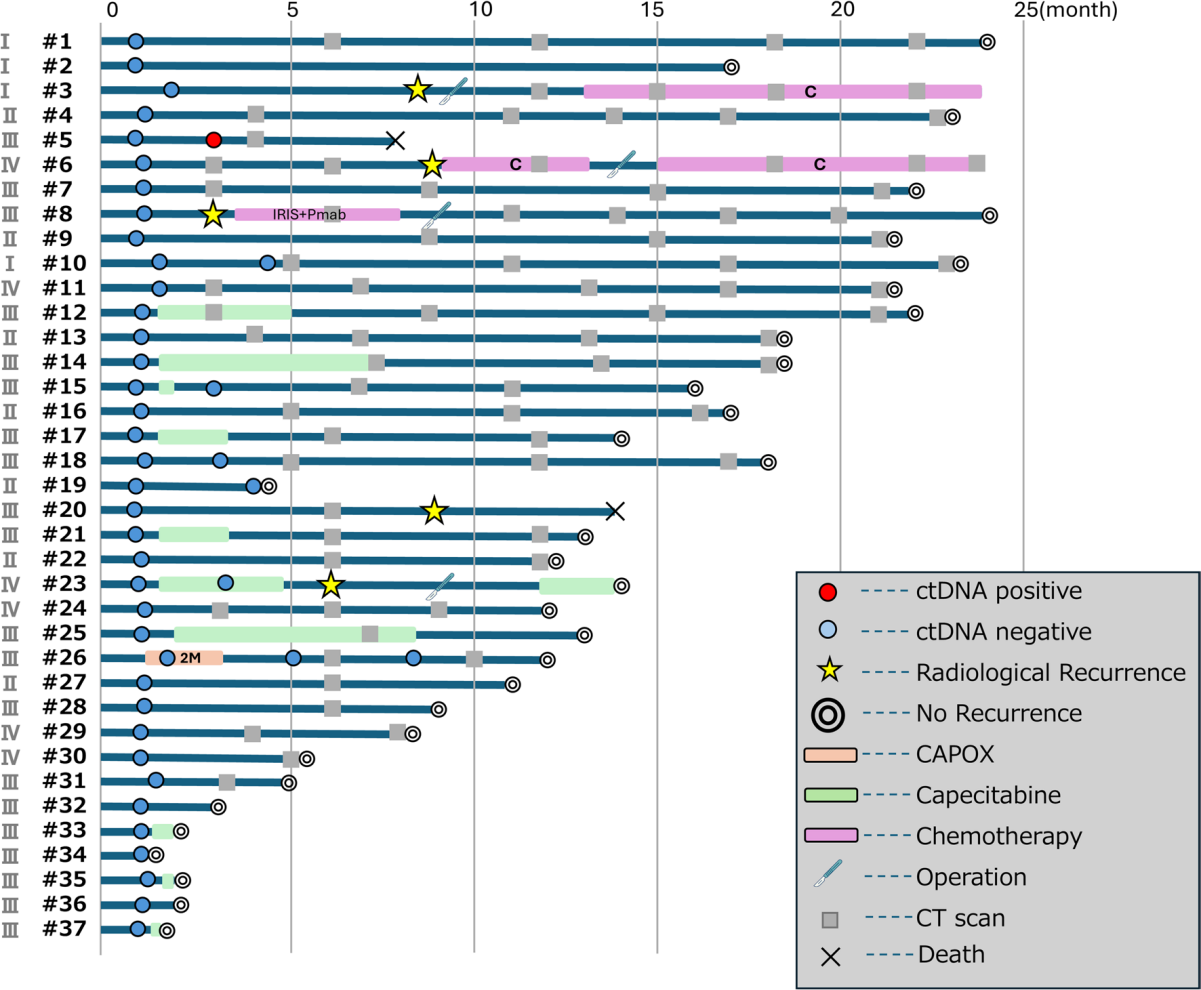
Details of ctDNA-negative patients (*n* = 37)

Among the 38 patients with postoperative ctDNA negativity, 37 were available for follow-up. The distribution of pathological stage was I/II/III/IV: 3 (8.1%)/8 (21.6%)/19 (51.4%)/7 (18.9%), with median follow-up of 13 months (range 1–24 months). All 3 pStage I patients did not receive adjuvant chemotherapy and have remained disease-free to date. Among the 8 pStage II patients, none received adjuvant chemotherapy. One patient (#3;12.5%) developed pulmonary metastasis and was treated with systemic chemotherapy, while the other 7 (#4, 9, 13, 16, 19, 22, 27; 87.5%) have not had recurrence. In the 19 pStage III patients, 9 (#5, 7, 18, 20, 28, 31, 32, 34, 36; 47.4%) underwent observation only, 9 (#12, 14, 15, 17, 21, 25, 33, 35, 37; 47.4%) received capecitabine monotherapy, and 1 (#26; 5.3%) received CAPOX. Of those under observation, 1 (#5; 11.1%) died of a non-cancer cause, and the other 8 (88.9%) have remained disease-free. Among the patients treated with capecitabine alone, 1 (#23; 11.1%) developed peritoneal dissemination; the rest (88.9%) have had no recurrence. The patient who received CAPOX also remains recurrence-free. Of the 7 pStage IV patients, 6 (#6, 8, 11, 24, 29, 30; 85.7%) were followed without adjuvant therapy and 1 (#23; 14.3%) received capecitabine monotherapy. 2 patients (#6, 8; 33.3%) developed recurrence (lymph node and lung metastasis, respectively) at 9 months after curative-intent surgery and are currently receiving systemic chemotherapy. The other 4 (66.7%) have not relapsed. The patient treated with capecitabine (#23) developed pulmonary recurrence and is receiving systemic therapy. For patients with negative postoperative ctDNA results, de-escalation to capecitabine monotherapy or observation alone was administered, particularly in elderly patients. (Fig. 4).

#### Surveillance of ctDNA-negative cases

Among the 37 patients with ctDNA negativity, 6 underwent serial ctDNA assessments (#5, 10, 15, 18, 19, 23, 26 in Fig. 4). Case #5 underwent ctDNA testing twice: the first at 4 weeks and the second at 4 months after curative-intent surgery. The second test was positive; however, imaging studies performed in response to the positive ctDNA result did not reveal recurrence, and the patient was managed with continued surveillance. The patient died of a non-cancer-related cause 8 months after surgery. Case #23 received adjuvant capecitabine monotherapy. Postoperative ctDNA testing was performed at 4 weeks and again at 4 months following the initial curative-intent resection, with both results returning negative. However, at 6 months postoperatively, surveillance CT identified pulmonary metastasis. The patient subsequently underwent surgical resection of the metastatic lesion. Adjuvant chemotherapy was resumed thereafter, and the patient remains disease-free. Cases #10, #15, #18, #19, and #26 underwent ctDNA testing twice, and both results were negative. These patients have had no recurrence after the primary surgery.

#### Recurrence sites—ctDNA-positive vs. -negative

**Fig. 5:**
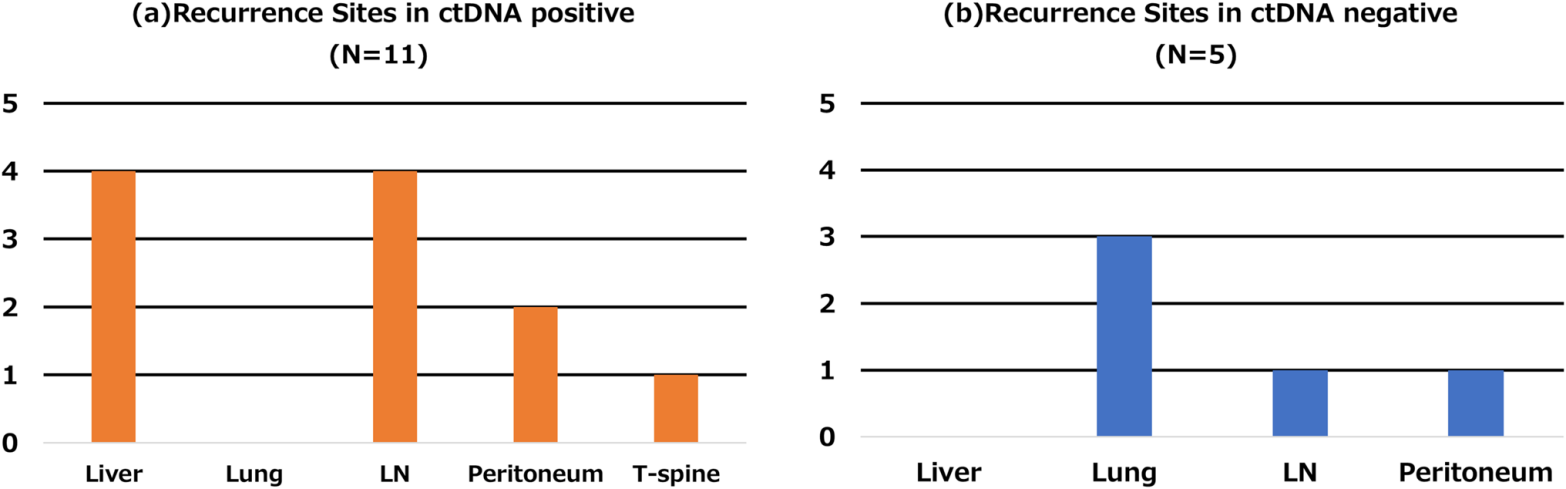
Recurrence sites based on ctDNA positivity: **a** ctDNA-positive cases (*N* = 11); **b** ctDNA-negative cases (*N* = 5)

Among the 16 patients with postoperative ctDNA positivity, recurrence was diagnosed based on ctDNA findings in 11 patients (positive predictive value 68.8%) (Fig. 5). Among these, 4 (36.4%) had liver metastases, 4 (36.4%) had lymph node metastases, 2 (18.2%) had peritoneal dissemination, and 1 (9.1%) had thoracic spine metastasis. No pulmonary metastasis was observed in this group. The median interval from curative-intent surgery to detection of liver metastasis was 2 months (range 0–3 months).

In contrast, among the 37 ctDNA-negative patients with follow-up, 5 (13.2%) were diagnosed with recurrence (negative predictive value 86.5%). Of these, 3 (60.0%) had pulmonary metastases, 1 (20.0%) had lymph node metastasis, and 1 (20.0%) had peritoneal dissemination. No liver metastases were detected in this group (Fig. 5). The median interval between curative-intent surgery and detection of lung metastasis was 6 months (range 3–10 months).

## Discussion

This real-world study demonstrated that postoperative ctDNA testing is a feasible, timely, and clinically informative tool for the postoperative management of patients with CRC. Our findings are consistent with previous reports in terms of positive and negative predictive value and sites of recurrence based on the ctDNA results [15, 16]. Among 56 patients who underwent ctDNA testing within 10 weeks after curative-intent surgery, 18 (32.1%) were ctDNA-positive, and 38 (67.9%) were ctDNA-negative. Radiological recurrence was subsequently observed in 62.5% of ctDNA-positive patients (10/16), compared with only 13.5% (5/37) in the ctDNA-negative cohort. The median turnaround time for ctDNA testing was 9 days, allowing timely incorporation of results into postoperative decision-making before the initiation of adjuvant chemotherapy. To the best of our knowledge, this is the first real-world study in Japan evaluating the utility of ctDNA for minimal residual disease (MRD) assessment after curative-intent surgery for CRC.

In this cohort, only one of 37 ctDNA-negative patients received CAPOX, whereas six of seven ctDNA-positive patients received CAPOX, tailoring adjuvant therapy to target only high-risk patients at the molecular level. In clinical practice, ctDNA testing provides substantial value for personalized postoperative management. For ctDNA-positive patients, it enables early imaging and treatment escalation, while for ctDNA-negative patients—particularly those who are elderly or frail and ineligible for clinical trials—it supports safe de-escalation of adjuvant therapy, reducing overtreatment without compromising outcomes. Two recently reported randomized trials incorporating ctDNA-guided strategies failed to meet their primary endpoints (recurrence-free survival) in stage II and III CRC [17, 18] despite decreased use of ACT, especially oxaliplatin. Further studies with larger multicenter cohorts and longer follow-up are warranted to refine ctDNA-driven treatment approaches. Collectively, our findings support the incorporation of ctDNA testing as a complementary biomarker alongside standard surveillance protocols in the postoperative management of CRC.

This study also underscores the clinical utility of serial ctDNA testing in selected cases. Longitudinal monitoring captured conversion from positive to negative ctDNA status, which correlated with sustained remission. Notably, among ctDNA-positive patients who received adjuvant CAPOX chemotherapy, 50.0% (3/6) achieved ctDNA clearance and remained recurrence-free during follow-up, supporting the potential role of ctDNA clearance as a surrogate marker of treatment efficacy as previously reported [9, 11]. Integrated analysis is ongoing to validate ctDNA clearance as a surrogate endpoint in ctDNA-positive CRC patients. Early detection of recurrence can lead to early intervention of treatment, which may prolong a patient's survival. In the GALAXY study, the lead time interval of ctDNA positivity to radiographic recurrence in resected CRC patients was 142 days [19]. In our cohort, among 16 patients with ctDNA detected within 10 weeks of curative-intent surgery, 9 had radiological evidence of metastatic disease. Moreover, the recurrence patterns differed by ctDNA status: liver metastasis was observed exclusively in the ctDNA-positive group, while lung metastasis occurred only among patients without ctDNA detected. Similar findings were reported in the GALAXY and COSMOS CRC studies, which demonstrated higher ctDNA detection rate in cases with liver metastasis recurrence compared to those with lung recurrence [16, 20]. This difference may reflect both biological and technical factors [21]. Liver metastasis is typically associated with higher tumor burden and greater ctDNA shedding due to abundant hepatic blood flow, whereas lung lesions may release smaller amounts of ctDNA because they must first return to the heart then travel through the arterial system before reaching the peripheral venous system [22]. In addition, the relatively small number of recurrence events in this interim analysis may also contribute to this observation. Several patients without ctDNA detected went on to develop recurrence (13.5% in this cohort,). This highlights a critical limitation of postoperative ctDNA testing: ctDNA negativity indicates lower recurrence risk but does not guarantee the absence of recurrence, particularly for lung and peritoneal metastases. Consequently, even ctDNA-negative patients require vigilant surveillance, and ctDNA results should be interpreted as one component of comprehensive postoperative monitoring rather than a definitive predictor of recurrence. Therefore, a single postoperative ctDNA assessment may not be sufficient to capture all recurrence patterns. Longitudinal ctDNA monitoring after curative-intent treatment could improve sensitivity for diverse recurrence sites and facilitate earlier clinical intervention for metastatic disease.

This study has several limitations. First, it was conducted at a single institution with a relatively small sample size, and the median follow-up duration in this cohort (13 months) may not be sufficient to capture late recurrences, particularly among ctDNA-negative patients. Since recurrences often occur beyond 2–3 years after surgery in pMMR CRC, the current dataset may overrepresent early recurrences. Second, because ctDNA testing was self-funded, selection bias may have occurred, favoring patients with higher socioeconomic status or stronger motivation for intensive surveillance. Third, adjuvant treatment decisions, including the use of chemotherapy, were left to the discretion of individual physicians rather than standardized protocols, which may have confounded outcome interpretation. Nevertheless, these real-world data provide valuable insights bridging the gap between controlled clinical trials and everyday clinical practice.

In conclusion, postoperative ctDNA testing appears to be a feasible and informative approach in real-world clinical practice. The present findings may facilitate early detection of recurrence and supports adjuvant chemotherapy decision-making after curative-intent surgery for CRC. In ctDNA-positive patients, early imaging and systemic treatment may help optimize disease management, whereas ctDNA-negative patients may benefit from treatment de-escalation strategies under careful surveillance. These exploratory observations indicate the potential role of ctDNA testing as a complementary biomarker in the postoperative management of colorectal cancer.
